# Dopamine versus norepinephrine in septic shock: a meta-analysis

**DOI:** 10.1186/cc9509

**Published:** 2011-03-11

**Authors:** S Shenoy, A Ganesh, A Rishi, V Doshi, S Lankala, J Molnar, S Kogilwaimath

**Affiliations:** 1 Rosalind Franklin University of Medicine and Science, North Chicago, IL, USA; 2 Memorial University of Newfoundland, St John's, Canada

## Introduction

The aim of this meta-analysis is to compare the changes in hemodynamic parameters among patients with septic shock who have received either of the two agents in their management and try to deduce the superiority of one over the other.

## Methods

A total of 880 articles were identified by a computerized search using MEDLINE, OVID and the Cochrane Central Register of Controlled Trials, of which six randomised controlled studies were included in the study. Observational data, retrospective studies or animal-based studies were excluded. Main outcome measures evaluated were the changes from the baseline in heart rate, mean arterial pressure, oxygen delivery index, oxygen extraction, systemic vascular resistance index (SVRI), cardiac index (CI), central venous pressure, blood lactate levels, urine output, mean pulmonary artery pressure (MPAP), pulmonary capillary wedge pressure, right ventricular ejection fraction (RVEF), arrhythmias and 28-day mortality rates. The statistical analysis was performed using Comprehensive Meta-Analysis software.

## Results

No significant difference was found in mortality between the two groups (RR = 1.067, CI = 0.984 to 1.157, *P *= 0.115). In the norpinephrine group, heart rate was significantly lower in comparison with baseline (mean change = -16.32 beats/minute, CI = -22.23 to -10.31, *P *< 0.001) and so also was the occurrence of arrhythmias (RR = 2.34, CI = 1.456 to 3.775, *P *< 0.001). The SVRI, however, was significantly higher in this group (difference in mean 185 dynes/cm^5^m^2^, CI = 141.214 to 229.05, *P *< 0.001). Patients who were on dopamine had significantly better RVEF% (mean difference = 2.38%, CI = 1.058 to 3.671, *P *< 0.001) and a lower lactate level (mean difference = -0.170 mmol/l, CI = -0.331 to -0.009, *P *= 0.038). Urine output, oxygen delivery, MPAP and oxygen consumption were not significantly different between the two groups.

## Conclusions

Patients who received dopamine had a better right ventricular ejection fraction, lower lactate levels, lower systemic vascular resistance index and a trend towards a better cardiac index. However, this group was noted to have more arrhythmias and a higher baseline heart rate versus the norepinephrine group. Overall, there was no difference in the 28-day mortality between the two groups. Although there are certain hemodynamic advantages, we were unable to deduce the superiority of one pressor. The results support the current practice of individualizing the choice of an initial vasopressor based on patient profile.

